# Deciphering the malignant synergy: metabolic reprogramming, epigenetic circuitry, and niche remodeling in osteosarcoma-macrophage crosstalk

**DOI:** 10.3389/fimmu.2026.1885433

**Published:** 2026-08-10

**Authors:** Zhi Zheng

**Affiliations:** Department of Orthopedics, Chengdu Integrated TCM & Western Medicine Hospital, Chengdu, China

**Keywords:** epigenetic circuitry, metabolic reprogramming, osteosarcoma, pre-metastatic niche, tumor-associated macrophages

## Abstract

Osteosarcoma (OS) prognosis remains stagnant due to the complex tumor microenvironment. This review deconstructs the “malignant synergy” between OS cells and tumor-associated macrophages (TAMs). Beyond the traditional M1/M2 dichotomy, we highlight the functional heterogeneity of TAMs, tracking their evolution from resident defenders to pro-metastatic accomplices. We elucidate three critical mechanisms driving this domestication: (1) Metabolic Reprogramming, where OS cells enforce immunosuppression via amino acid and lipid hijacking; (2) Epigenetic Circuitry, involving m6A modifications and ceRNA networks that create self-reinforcing malignant loops; and (3) Niche Remodeling, where exosomal fusion proteins remotely condition the lung for metastasis. By synthesizing these mechanisms, we identify key vulnerabilities in immune evasion and chemoresistance, providing a rationale for therapies targeting this critical interaction.

## 1 Introduction

Osteosarcoma (OS) represents the most aggressive and prevalent primary malignant bone tumor, characterized by a bimodal age distribution with an initial peak in children—where it arises as a primary bone neoplasm—and a second peak in adults, typically associated with prior radiation exposure or underlying disorders. The disease predominantly affects the long bones of the lower extremities and exhibits a strong propensity for distant dissemination, particularly to other skeletal sites and the lungs (1–3). Notably, at initial diagnosis, approximately 20% of patients already present with metastatic lesions, and nearly 90% of these involve pulmonary metastases (4). Although OS is relatively rare, with an incidence of 3–5 cases per million annually, it carries substantial morbidity and mortality (5). Around 60% of affected individuals are adolescents aged 10–20 years, making OS the second leading cause of cancer-related death in this population (1). The current therapeutic paradigm—neoadjuvant chemotherapy followed by surgical resection and subsequent adjuvant chemotherapy—has markedly improved outcomes for patients with localized disease. Nevertheless, the five-year survival rate remains 60–70% for localized OS but falls below 20% for those with metastatic disease (6). Due to the pronounced genomic instability and molecular heterogeneity of OS, conventional therapies that primarily target tumor cells have reached significant limitations.

In recent years, research priorities have shifted from a “tumor cell–centric” paradigm toward a systematic deconstruction of the tumor microenvironment (TME). A prevailing consensus now holds that the malignant phenotype of OS arises from the coordinated outcome of intricate information exchange between tumor cells and the surrounding stromal and immune compartment. Among the diverse immune constituents infiltrating OS, tumor-associated macrophages (TAMs) represent the most abundant population within the TME (7, 8). The traditional dichotomous framework that classifies macrophages into antitumoral M1 and protumoral M2 subsets has proven overly simplistic (9, 10). With the advent of single-cell RNA sequencing (scRNA-seq), TAMs in OS have been revealed to possess far greater heterogeneity and functional plasticity than previously appreciated. Subpopulations ranging from C1Q^+^ TAMs (11), which reside in the primary lesion and help maintain immune homeostasis, to FABP4^+^ TAMs (5), which accumulate in pre-metastatic niches (PMN) and promote cellular migration, illustrate the refined “recruitment” and “conditioning” strategies employed by OS cells to shape the immune landscape. The mechanistic basis of this conditioning involves several evolutionarily honed biological processes. First is the diversification of communication mediators: beyond classical chemokine axes, OS cells deploy nanoscale vesicular carriers—including exosomes, migrasomes, and microparticles—to achieve long-range, highly specific delivery of proteins and non-coding RNAs (12–14). Second is deep metabolic coupling: by rewiring amino acid and lipid metabolic fluxes—such as L-amino acid transporter 2 (LAT2)-mediated leucine uptake or lysosomal acid lipase A (LIPA)-driven lipid accumulation—OS cells not only sustain their own biosynthetic demands but also impose “metabolic ligands” that forcibly reshape TAM immune polarization (15, 16). Finally, epigenetic regulation intertwines with signaling feedback: N6-methyladenosine (m6A) modifications and complex competing endogenous RNA (ceRNA) networks act in a mutually reinforcing manner, establishing multiple malignant positive-feedback loops between OS cells and macrophages that are difficult to disrupt (17–19).

This review dissects the intricate molecular crosstalk between OS cells and TAMs. We systematically navigate the multi-dimensional architecture of this partnership: from the ontogeny and spatial heterogeneity of TAMs to the ‘metabolic hijacking’ and epigenetic circuitry—specifically m6A modifications and ceRNA networks—that enforce the immunosuppressive phenotype. Furthermore, we emphasize the systemic reach of this alliance, elucidating how novel mediators, such as specific fusion proteins, orchestrate the remote conditioning of the pulmonary PMN. By integrating these physicochemical, molecular, and systemic mechanisms, our objective is to expose the core logic of this ‘malignant synergy,’ providing a theoretical foundation for disrupting the immune-evasive network in OS.

## 2 Macrophage heterogeneity and environmental shaping in OS

**Figure 1:**
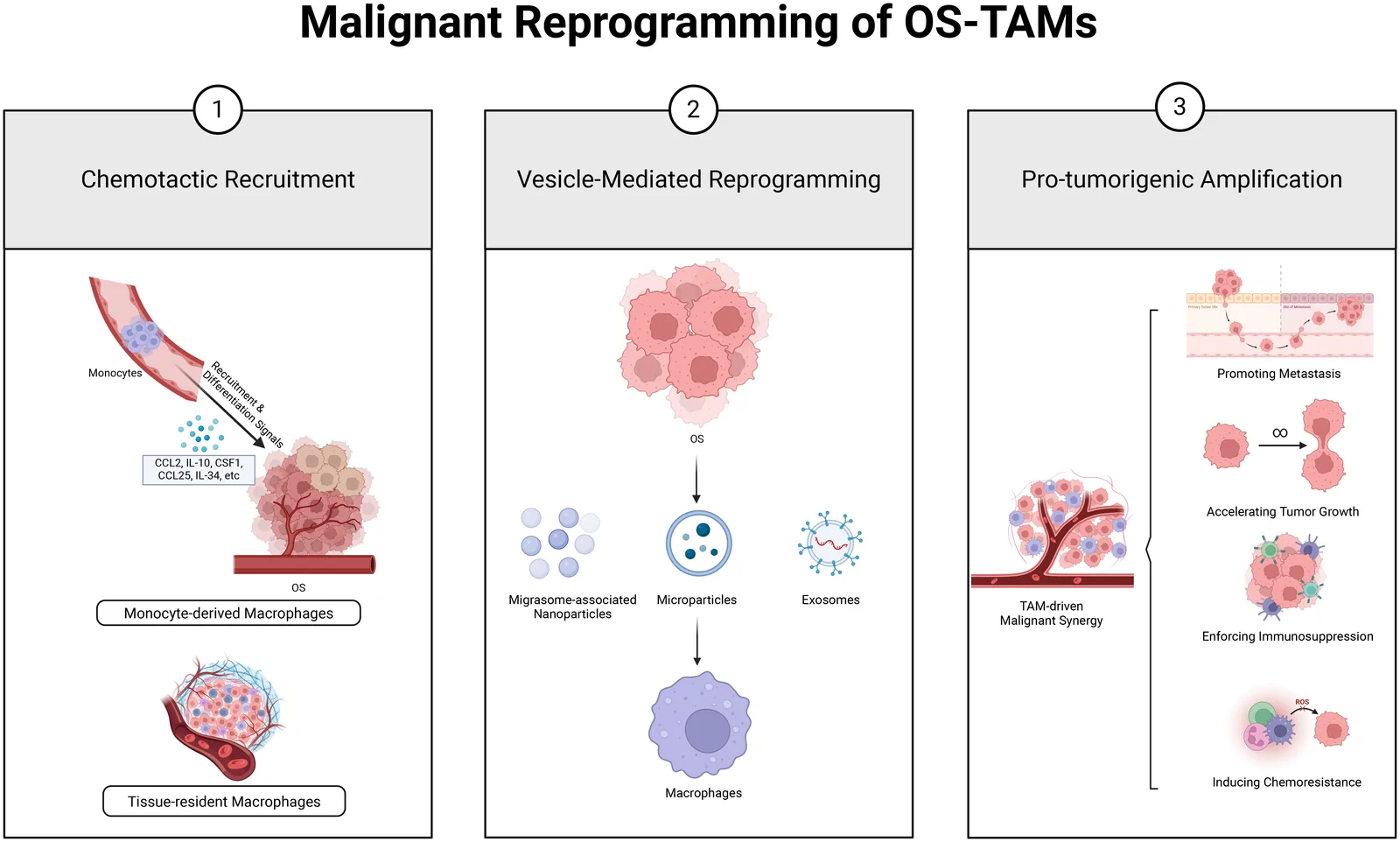
Malignant reprogramming of OS-TAMs. This figure illustrates the sequential process where OS cells actively recruit monocytes via chemotactic signals and reprogram them through vesicle-mediated delivery (migrasomes, exosomes, and microparticles). These domesticated TAMs establish a “TAM-driven malignant synergy” that actively drives disease progression by promoting metastasis, accelerating tumor growth, enforcing immunosuppression, and inducing chemoresistance. OS, Osteosarcoma; TAMs, Tumor-associated macrophages.

The research paradigm of macrophage biology has undergone a profound transformation over the past century. While early studies primarily characterized macrophages solely as phagocytes within the reticuloendothelial system, modern lineage tracing technologies have established the “dual-origin” theory. This theory posits that macrophages are derived not only from circulating monocytes differentiated from bone marrow hematopoietic stem cells but also from yolk sac progenitors during early embryonic development (20, 21). Notably, these tissue-resident macrophages colonize the bone microenvironment prenatally (**Figure 1**). This heterogeneity in origin lays the foundation for understanding their distinct functions within the TME (22).

Functionally, macrophages exhibit remarkable plasticity, with their phenotypes dependent on induction by microenvironmental signals. The classical binary classification categorizes them into classically activated M1 and alternatively activated M2 phenotypes. The former, stimulated by lipopolysaccharide or IFN-γ, exerts anti-tumor effects through the release of reactive oxygen species and pro-inflammatory cytokines (e.g., TNF-α) (23, 24). Conversely, the latter is polarized by IL-4 or IL-13 to mediate immunosuppression and tissue repair, thereby fostering tumor progression (25). However, under complex pathological conditions within tumors, TAMs often exhibit a continuous spectrum of activation that transcends the simplistic “M1-M2” dichotomy. This includes various subtypes, such as M2a, M2b, and M2c, as well as mixed phenotypes (26). This high degree of dynamic plasticity constitutes the cellular basis for tumor immune evasion.

Facilitated by breakthroughs in scRNA-seq, our understanding of the heterogeneity of TAMs in OS has transcended the limitations of surface markers, extending deeply into the dimension of functional subsets. Within single-cell atlases of advanced and metastatic OS, unbiased clustering analysis has revealed significant spatial and functional stratification among TAMs. Notably, a distinct population of C1Q+ macrophages, characterized by high expression of complement genes (C1QA, C1QB, C1QC), has been identified as a primary anti-tumor immune subpopulation within the TME. The infiltration abundance of this subset is positively correlated with favorable patient prognosis in the TARGET-OS cohort (11), representing the residual force of “immune surveillance” within the microenvironment (11). In contrast, FABP4+ macrophages were discovered in a single-cell RNA-seq cohort of advanced primary and pulmonary metastatic OS tissues (5). These cells exhibit active pro-inflammatory characteristics; the emergence of this subpopulation is frequently associated with heightened invasiveness and immunosuppressive capabilities (5). Beyond traditional single-cell transcriptomic profiling, spatially resolved transcriptomic atlases of human osteosarcoma have mapped the structural organization of TAM subsets within native tumor tissues (27). These studies reveal spatial co-localization patterns between specific myeloid/TAM subpopulations and malignant osteosarcoma cells in clinical specimens, supporting the formation of localized niches that contribute to an immunosuppressive tumor microenvironment.

The heterogeneity and functional plasticity of TAMs are not predetermined but are dynamically shaped by their local microenvironment. Physicochemical factors, particularly the 3D extracellular matrix and oxygen tension, play pivotal roles in this process. Physically, the bone marrow microenvironment provides a unique 3D spatial structure; studies indicate that compared to 2D monolayer cultures, TAMs in 3D systems exhibit heightened sensitivity to tumor signals, suggesting that such spatial physical confinement is a prerequisite for maintaining their pro-tumorigenic phenotype (28). Chemically, hypoxia induced by rapid tumor growth acts as a critical metabolic switch. Under hypoxic conditions, M2 macrophages are induced to secrete substantial amounts of osteopontin, which accelerates tumor progression by downregulating ISG15 and EGR3 while activating the RIG-I pathway (29). This synergy between physical support and chemical induction constitutes the environmental foundation for the reprogramming of TAMs by OS.

## 3 Malignant domestication: recruitment and polarization

OS progression depends not merely on the cell-autonomous traits of tumor cells, but critically on their capacity to sculpt an immunosuppressive microenvironment, wherein the “education” of macrophages constitutes a pivotal element. This process initiates with systemic cell recruitment and drives functional polarization through complex intercellular crosstalk, ultimately transforming macrophages into accomplices of tumor progression.

### 3.1 Chemotactic recruitment and positioning

The infiltration of macrophages into the tumor mass is dictated by specific chemotactic gradients established by OS cells (**Figure 1**). While the CCL2-CCR2 axis is classically recognized as the primary driver of monocyte recruitment, recent evidence suggests that OS employs a more complex, context-dependent network to modulate immune infiltration.

The regulation of CCL2 involves intricate upstream mechanisms. Spondin-2 (SPON2), an extracellular matrix protein significantly upregulated in OS (30, 31), has been identified as a key driver of this axis (30). SPON2 activates the NF-κB/VEGF signaling pathway, thereby enhancing the secretion of CCL2, IL-10, and CSF1. This cytokine cocktail not only recruits monocytes but also actively promotes their polarization toward an M2-like phenotype, fostering an immunosuppressive and angiogenic niche (30). Conversely, the loss of tumor suppressors plays a crucial role in amplifying these signals. For instance, tumor necrosis factor-α-induced protein 8-like 1 normally acts as a brake on inflammation; its downregulation in OS cells relieves the suppression of CCL2, leading to uncontrolled macrophage infiltration and accelerated tumor growth (32).

Beyond the primary bone lesion, macrophage recruitment to lung metastases requires specific molecular signals. Recent evidence identifies Zinc finger imprinted 3 (ZIM3) as a key driver of this process in osteosarcoma: ZIM3 transcriptionally upregulates CCL25 to draw macrophages to the lung parenchyma (33). Canonically, CCL25 signals through the CCR9 receptor to recruit M2 macrophages. While direct evidence for CCR9 dependency in OS remains to be fully elucidated, the CCL25/CCR9 axis is a well-documented conduit for macrophage recruitment in other lung-resident tumors (34), as well as in melanoma (35) and non-alcoholic steatohepatitis (36). Recruited via this broadly conserved axis, these macrophages are rapidly polarized into M2 subsets within the lung, aiding the colonization of disseminating tumor cells. This ZIM3-CCL25 axis highlights the spatial specificity of macrophage recruitment, distinguishing the metastatic niche from the primary TME (33).

Furthermore, the recruitment process is often coupled with angiogenesis. IL-34, a cytokine functionally akin to M-CSF, serves as a dual-function mediator. Regulated by pro-inflammatory signals like TNF-α and IL-1β, IL-34 not only recruits M2-polarized TAMs but also directly promotes the growth of endothelial cells and the assembly of vascular structures (37). This suggests that OS cells coordinate macrophage recruitment synchronously with neovascularization to ensure metabolic support for the expanding tumor mass.

### 3.2 Vesicular communication carriers

While soluble cytokines mediate diffusion-based signaling, OS cells also deploy membrane-bound carriers for the delivery of complex, high-density molecular information. These carriers—including migrasomes, microparticles, and exosomes—enable the transfer of proteins and nucleic acids that might otherwise be unstable in the extracellular space, functioning as potent “reprogramming packages” for TAMs (**Figure 1**).

Migrasomes, recently discovered organelles formed during cell migration, represent a novel mechanism of tumor-immune crosstalk (38). In OS, migrating tumor cells leave behind migrasome-associated nanoparticles (MANPs) enriched with MFGE8 (milk fat globule-EGF factor 8). These MANPs act as “nutritional traps” that are eagerly phagocytosed by surrounding macrophages. Upon internalization, MFGE8 drives a potent M2 polarization program, thereby converting the immune stroma into a supportive track for tumor migration (13). This finding is particularly significant as it links the physical act of tumor cell migration directly to immune modulation.

Similarly, tumor-derived microparticles (T-MPs) serve as vectors for oncogenic signaling. Derived from the plasma membrane, T-MPs shed by OS cells have been shown to activate the TBK1-STAT6 signaling axis in recipient macrophages. This activation forces macrophages into an M2-like state, characterized by the secretion of CCL18 (12). In a malignant feed-forward loop, CCL18 then acts back on the tumor cells via STAT3 signaling to enhance migration and induce chemoresistance, demonstrating how T-MPs establish a bidirectional symbiosis between the tumor and immune compartments (12).

Finally, exosomes function as nanoscale vectors for molecular reprogramming. OS-derived exosomes have been implicated in facilitating lung metastasis by inducing the expression of Tim-3 on macrophages. The uptake of these exosomes triggers a phenotypic switch that suppresses immune surveillance and prepares the PMN for colonization (14). Despite their potent biological roles, the functional interpretation of exosomal conditioning is currently hindered by methodological bottlenecks. The OS literature frequently displays significant heterogeneity in vesicle isolation techniques (e.g., ultracentrifugation versus commercial size-exclusion chromatography). Such variations risk the co-isolation of non-exosomal proteins and protein aggregates, confounding the precise attribution of metastatic conditioning to exosomes (39). To ensure reproducibility in OS-TAM crosstalk research, strict adherence to standardized isolation and characterization protocols, such as the Minimal Information for Studies of Extracellular Vesicles (MISEV) guidelines, is urgently required in future human validation cohorts (40). Collectively, these vesicular transport systems—migrasomes, microparticles, and exosomes—illustrate the sophistication of OS cells in utilizing diverse physical carriers to ensure the precise and robust delivery of protumorigenic instructions to the immune microenvironment.

## 4 Metabolic reprogramming: the nutritional logic of immunosuppression

The metabolic landscape of the OS microenvironment is characterized by fierce nutrient competition and intricate metabolic coupling. Tumor cells do not merely deplete nutrients; they actively reprogram the metabolic fluxes of amino acids, lipids, and glucose to impose metabolic checkpoints on immune cells. This “metabolic hijacking” forces TAMs to abandon their antitumor functions and adopt an M2-like metabolic program that supports tumor survival and evasion.

### 4.1 Amino acid metabolism: the biosynthetic and signaling nexus

**Figure 2:**
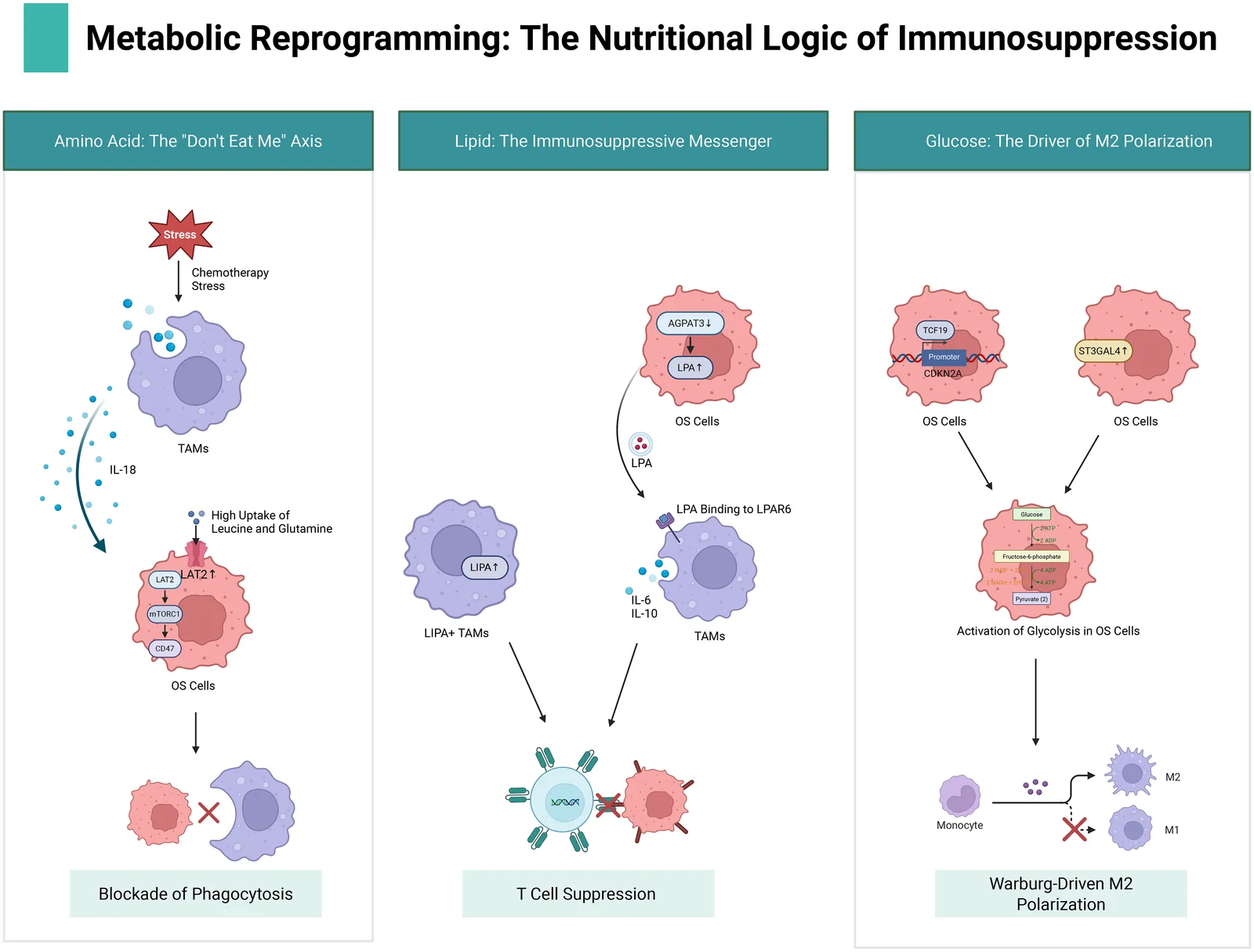
Metabolic reprogramming: The nutritional logic of immunosuppression. In the amino acid axis (left), chemotherapy-induced IL-18 from TAMs promotes LAT2-mediated leucine and glutamine uptake in OS cells, activating the mTORC1 pathway to drive CD47 expression and effectively block phagocytosis. In the lipid axis (middle), the overexpression of LIPA and downregulation of AGPAT3 in OS cells synergistically suppress T cell activity. In the glucose axis (right), genetic (TCF19/CDKN2A) and metabolic (ST3GAL4) regulators coordinate to reinforce the Warburg effect in OS cells, creating a glycolytic microenvironment that enforces M2 polarization of TAMs. OS, Osteosarcoma; TAMs, Tumor-associated macrophages; LAT2, L-amino acid transporter 2; LIPA, Lysosomal acid lipase A; AGPAT3, 1-acylglycerol-3-phosphate O-acyltransferase 3; LPA, Lysophosphatidic acid; ST3GAL4, ST3 beta-galactoside alpha-2,3-sialyltransferase 4.

Amino acids serve not only as biosynthetic building blocks but also as critical signaling molecules that regulate immune recognition (**Figure 2**). In the context of chemotherapy, OS cells orchestrate a sophisticated adaptive mechanism centered on amino acid transport. Recent findings reveal that chemotherapy stress induces macrophages to secrete IL-18. This cytokine acts on tumor cells to upregulate the LAT2, leading to a massive influx of leucine and glutamine (16). This surge in amino acid uptake has profound immunological consequences. Leucine and glutamine are potent activators of mTORC1. The hyperactivation of the mTORC1 pathway subsequently drives c-Myc-mediated transcription of CD47—a crucial “don’t eat me” signal (41, 42). Consequently, this metabolic axis (IL-18/LAT2/mTORC1/CD47) effectively allows OS cells to evade phagocytosis by macrophages, highlighting how amino acid metabolism is directly rewired to orchestrate immune escape (16). Targeting LAT2 has been shown to disrupt this axis, sensitizing tumors to doxorubicin.

Furthermore, the broader Solute Carrier (SLC) family plays a pivotal role in this landscape. SLC7A1, another cationic amino acid transporter (43, 44), exhibits elevated expression levels specifically within OS cells, which is associated with adverse clinical outcomes. Single-cell profiling indicates that SLC7A1 expression is not merely a marker of proliferation but a functional modulator of the macrophage niche, driving M2 polarization through intercellular crosstalk (45). Collectively, these transporters transform the TME into an amino acid-depleted zone for immune cells while fueling the tumor’s defensive signaling.

### 4.2 Lipid metabolism: remodeling the lipidomic profile

Lipid metabolism in OS extends beyond energy storage, serving as a driver of immunosuppressive signaling (**Figure 2**). The impact of systemic lipid availability is evident, as high-fat diets have been observed to accelerate tumor progression and lung metastasis. Central to this process is LIPA, a critical regulator of intracellular lipid hydrolysis (46). Elevated LIPA expression is identified as an independent risk factor associated with poor survival. Mechanistically, LIPA drives the reprogramming of macrophages into a distinct immunosuppressive subset (15). These LIPA-high macrophages facilitate T cell suppression and promote tumor progression, establishing a lipid-dependent immune tolerance (15). Notably, pharmacological inhibition of LIPA (e.g., using Glecaprevir in preclinical models) has been shown to reverse this phenotype, suggesting that targeting lipid hydrolysis is a viable therapeutic strategy.

Conversely, alterations in glycerolipid metabolism can also unleash immunosuppressive mediators. 1-acylglycerol-3-phosphate O-acyltransferase 3 (AGPAT3) typically functions as a tumor suppressor (47); its downregulation correlates with poor outcomes. The loss of AGPAT3 in OS cells leads to the accumulation of lysophosphatidic acid (LPA) (48). This bioactive lipid acts as an intercellular messenger, binding to LPAR6 on TAMs. The activation of the LPA-LPAR6 axis stimulates TAMs to secrete IL-6 and IL-10, cytokines that potently inhibit the cytotoxic function of CD8+ T cells (48). Thus, both the upregulation of lipolytic enzymes (LIPA) and the dysregulation of lipid synthesis (AGPAT3) converge to shape a lipid-rich, immunosuppressive microenvironment.

### 4.3 Glucose metabolism and metabolic stress

The “Warburg effect” is a hallmark of OS, but its impact transcends simple energy production; it fundamentally alters the immune state via metabolite accumulation and metabolic stress (**Figure 2**). High rates of glycolysis in tumor cells create a glucose-deprived, lactate-rich environment that favors M2 polarization. This glycolytic switch is tightly regulated by genetic and epigenetic factors. TCF19 has been shown to bind the promoter of CDKN2A, enhancing its expression. Upregulated CDKN2A drives the glycolytic flux in OS cells, which, in turn, modulates macrophage plasticity—specifically decreasing M1 markers (CD86) while increasing M2 markers (CD206) and IL-10 secretion (49).

Crucially, recent multi-omics analyses have expanded our view beyond glucose to encompass vitamin and cofactor metabolic pathways, identifying them as key prognostic regulators of the tumor immune microenvironment. Metabolic clustering reveals that variations in these pathways significantly impact immune cell infiltration and chemotherapy response (50, 51). Central to this network is ST3 beta-galactoside alpha-2,3-sialyltransferase 4 (ST3GAL4), identified as a key risk gene. ST3GAL4 does not only drive the malignant proliferation and invasion of OS cells but also fundamentally rewires their glycolytic capacity (52). Knockdown of ST3GAL4 significantly dampens glycolysis and, importantly, reverses the M2 polarization of co-cultured macrophages (52). This suggests that sialylation and cofactor metabolism are inextricably linked to the glycolytic control of immune surveillance.

Finally, metabolic stress manifests in novel forms of cell death, such as disulfidptosis. The disulfidptosis-associated long non-coding RNA, LINC01137, has been identified as a critical stress-response mediator (53, 54). Elevated LINC01137 expression not only enhances epithelial-mesenchymal transition (EMT) but also modulates macrophage orientation within the TME, linking disulfide stress handling directly to immune escape mechanisms (55).

## 5 Molecular circuitry: epigenetics, ceRNA networks, and reciprocal feedback

**Figure 3:**
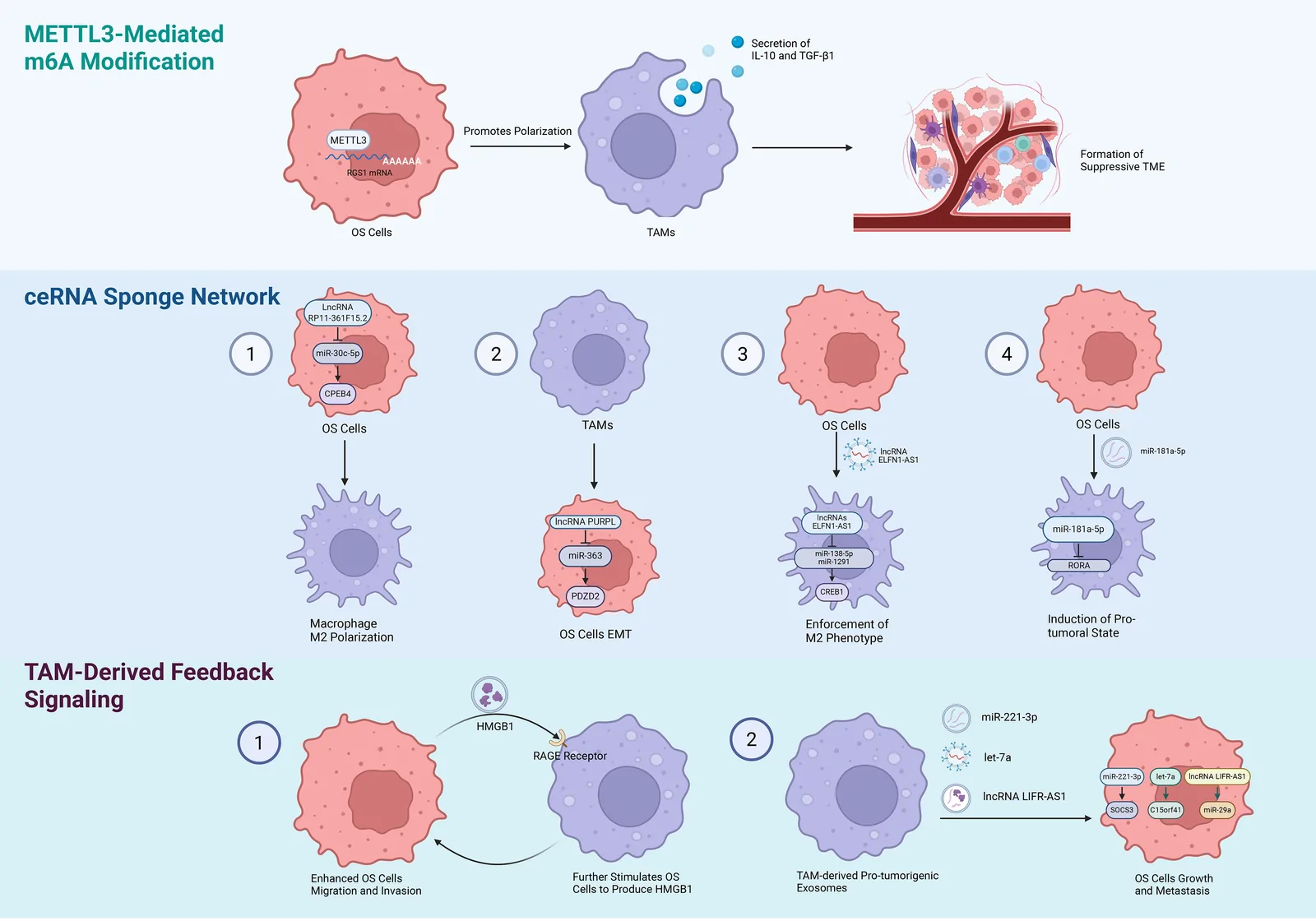
Molecular circuitry of OS-TAM crosstalk. This figure delineates the multi-layered regulatory networks governing the malignant synergy. In the epigenetic axis (top), METTL3 stabilizes RGS1 mRNA via m6A modification in OS cells, driving M2 polarization and the formation of a suppressive TME via IL-10/TGF-β1 secretion. The ceRNA sponge network (middle) integrates intracellular (RP11-361F15.2 and PURPL) and exosome-mediated intercellular (ELFN1-AS1 and miR-181a-5p) mechanisms, functioning as molecular switches to enforce macrophage M2 phenotypes and promote OS cell EMT. Finally, TAM-derived feedback signaling (bottom) establishes self-reinforcing loops via the HMGB1-RAGE axis and pro-tumorigenic exosomes (carrying miR-221-3p, etc.), which suppress tumor suppressors like SOCS3 to accelerate OS growth and metastasis. OS, Osteosarcoma; TAMs, Tumor-associated macrophages; ceRNA, Competing endogenous RNA; EMT, Epithelial-mesenchymal transition; m6A, N6-methyladenosine; RGS1, Regulator of G-protein signaling 1; lncRNAs, Long non-coding RNAs; CPEB4, Cytoplasmic polyadenylation element binding protein 4; METTL3, Methyltransferase-like 3.

While metabolic reprogramming provides the fuel for tumor progression, the instruction manual is written by a complex network of genetic and epigenetic regulators. In OS, the crosstalk between tumor cells and macrophages is not random but strictly governed by m6A modifications, ceRNA networks, and self-reinforcing feedback loops (**Figure 3**).

Epigenetic modifications, particularly RNA methylation, have emerged as pivotal regulators of immune evasion (**Figure 3**). A recent study has elucidated a critical mechanism involving methyltransferase-like 3 (METTL3), a core m6A methyltransferase. In OS cells, METTL3 functions as a stabilizer for the mRNA of regulator of G-protein signaling 1 (RGS1) (19, 56, 57). By depositing m6A modifications onto RGS1 transcripts, METTL3 prevents their degradation, leading to sustained high levels of RGS1 protein. This stabilization has profound downstream effects: elevated RGS1 acts as an essential driver for the M2 polarization of macrophages (19). These polarized TAMs, in turn, secrete IL-10 and TGF-β1, creating a suppressive milieu that further enhances the tumorigenicity of OS cells (19). This “METTL3-RGS1-M2” axis highlights how an intracellular epigenetic event in tumor cells can dictate the fate of the extracellular immune environment. Targeting the m6A reader/writer machinery or RGS1 directly could therefore dismantle this fundamental layer of immune suppression.

Non-coding RNAs function as sophisticated molecular switches through the ceRNA mechanism. In this “sponge” model, long non-coding RNAs (lncRNAs) competitively bind to specific miRNAs, thereby preventing the degradation of downstream target mRNAs (**Figure 3**). However, it must be emphasized that current discoveries regarding specific lncRNAs in OS represent preliminary findings heavily dependent on specific experimental models, rather than universally validated mechanisms across all OS heterogeneities. For example, in *in vitro* and xenograft models, LncRNA RP11-361F15.2 has been shown to act as a decoy for miR-30c-5p, effectively liberating cytoplasmic polyadenylation element binding protein 4 (CPEB4) from repression (18). The accumulation of CPEB4 subsequently drives both tumor invasion and M2 macrophage polarization (18). Similarly, the lncRNA PURPL acts as a responder to TAM infiltration specifically within MG-63 cell co-culture systems; in this context, TAMs induce PURPL expression in tumor cells, which then sponges miR-363 to upregulate PDZD2, further promoting EMT (58). Crucially, this ceRNA regulation transcends cell boundaries via exosomes, although current evidence remains localized to specific cell lines. For instance, investigations utilizing 143B cells demonstrated that these OS cells package specific lncRNAs, such as ELFN1-AS1, into exosomes to be delivered into macrophages. Upon entry, ELFN1-AS1 sponges miR-138-5p and miR-1291, leading to the upregulation of CREB1 and enforcing an M2 phenotype (59). Conversely, tumor cells also utilize exosomes to deliver miRNAs like miR-181a-5p, which directly targets and suppresses RORA in macrophages, further skewing them toward a protumoral state (17). These findings illustrate a multi-layered regulatory network where coding and non-coding RNAs traverse the TME to synchronize tumor-immune interactions. However, translating these ceRNA networks into clinical applications requires extreme caution. The majority of these mechanisms are delineated in murine patient-derived xenograft (PDX) models or *in vitro* systems. Unlike protein-coding genes, lncRNAs exhibit notably low evolutionary conservation and high species specificity across mammals (60, 61). Consequently, the precise lncRNA-miRNA-mRNA nodes identified in murine OS models often lack direct structural orthologs in human tissues. Future studies must prioritize spatial transcriptomics on primary human OS specimens to validate which of these epigenetic circuits are genuinely active in patients, ensuring that potential therapeutic targets are not merely murine-specific artifacts.

The interaction between OS and macrophages is not a unidirectional instruction but a bidirectional, self-reinforcing cycle (**Figure 3**). Once polarized, M2 macrophages actively signal back to the tumor cells, often exacerbating malignancy through defined feedback loops. One such mechanism involves the HMGB1-RAGE axis. OS cells secrete HMGB1 to polarize macrophages via the RAGE receptor; in a positive feedback loop, these M2 macrophages stimulate the tumor cells to produce even more HMGB1, amplifying migration and invasion capabilities (62). Exosomes derived from TAMs serve as the primary carriers for this reverse signaling. M2-polarized TAMs secrete exosomes enriched with miR-221-3p, which are internalized by OS cells. This miRNA targets SOCS3, a negative regulator of cytokine signaling. The suppression of SOCS3 consequently unleashes the JAK2/STAT3 pathway, aggressively promoting tumor growth and metastasis (63). Similarly, TAM-derived exosomes carrying let-7a target C15orf41, while those carrying lncRNA LIFR-AS1 sponge miR-29a to upregulate NFIA (64, 65). In all cases, the macrophage—originally recruited as a defender—is educated to provide molecular signals that close a vicious cycle of disease progression.

## 6 The lung PMN: remote conditioning and colonization strategy

The “seed and soil” hypothesis, proposed by Stephen Paget over a century ago, finds its most critical application in OS, where pulmonary metastasis accounts for the vast majority of patient mortality (66). Current evidence suggests that the lung is not a passive recipient of metastasis; rather, it is actively “primed” by the primary tumor long before distinct metastases appear. This remote conditioning creates a PMN dominated by immunosuppressive macrophages.

### 6.1 Exosomal cargo and fusion proteins: architects of the PMN

**Figure 4:**
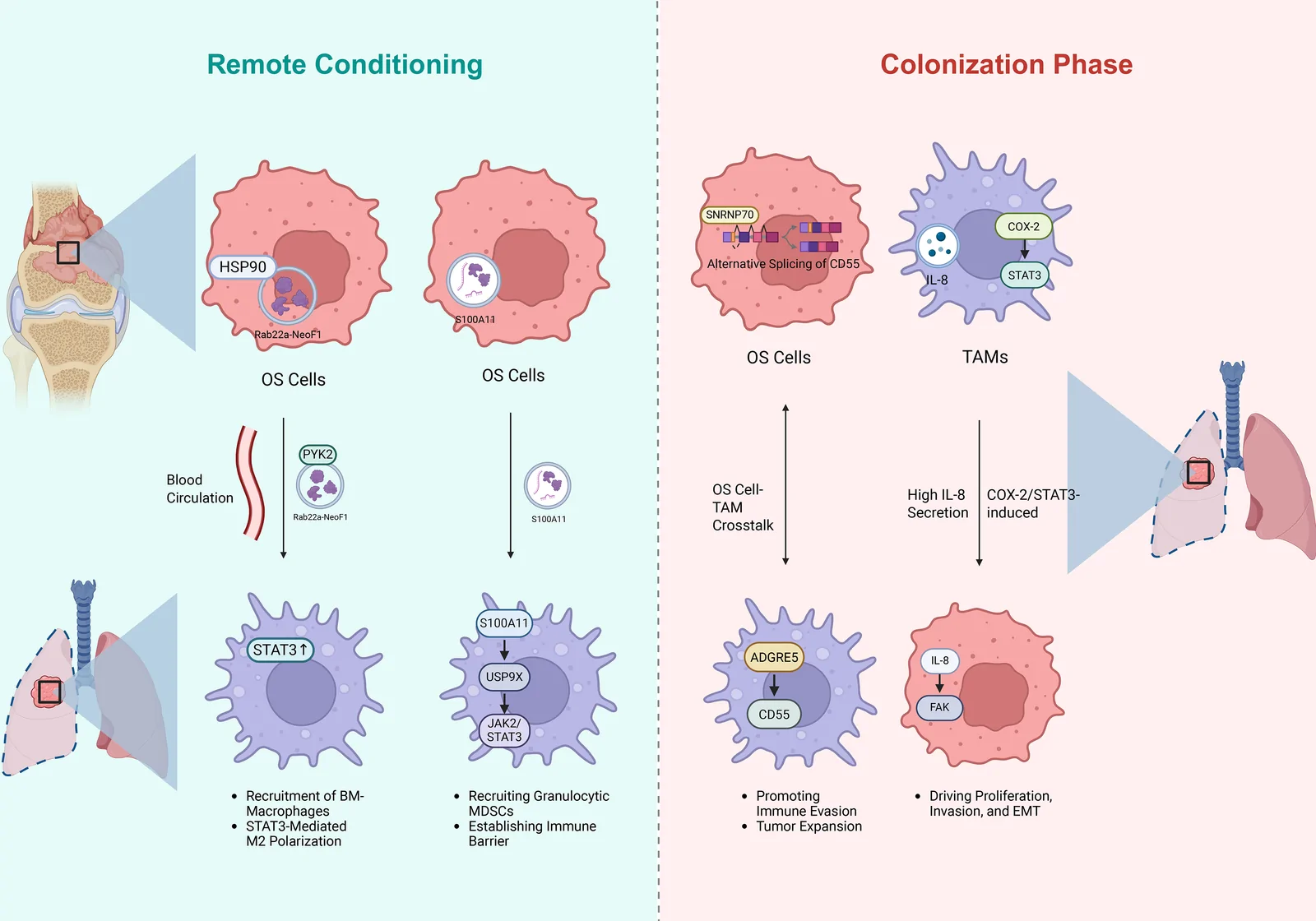
The lung PMN: remote conditioning and colonization strategy. This figure illustrates the spatiotemporal evolution of the metastatic niche. In the remote conditioning phase (left), primary OS cells utilize HSP90 to sort Rab22a-NeoF1 fusion proteins into exosomes, which recruit bone marrow-derived macrophages to the lung and induce STAT3-mediated M2 polarization; concurrently, S100A11-loaded EVs activate the USP9X/JAK2/STAT3 axis in pulmonary macrophages to recruit granulocytic MDSCs, establishing an immunosuppressive barrier. In the colonization phase (right), SNRNP70-mediated splicing of CD55 promotes ADGRE5-dependent immune evasion, while cytokine signaling (IL-8/FAK and COX-2/STAT3) drive proliferation, invasion, and EMT, collectively fueling aggressive metastatic expansion. OS, Osteosarcoma; TAMs, Tumor-associated macrophages; EMT, Epithelial-mesenchymal transition; PMN, Pre-metastatic niche; SNRNP70, U1 small nuclear ribonucleoprotein 70K; MDSCs, Myeloid-derived suppressor cells.

Exosomes serve as the primary long-distance messengers in this process (**Figure 4**). A groundbreaking study has recently identified a specific mechanism involving the Rab22a-NeoF1 fusion protein, a driver of OS malignancy (67). This fusion protein does not randomly enter exosomes; it is specifically sorted by HSP90 via a unique KFERQ-like motif (RVLFLN (142)). Upon secretion, exosomal Rab22a-NeoF1—complexed with its binding partner PYK2—travels to the lungs (68). There, it performs a dual function: it acts as a beacon to recruit bone marrow-derived macrophages to the lung parenchyma and simultaneously activates STAT3 signaling within these macrophages to enforce an M2 phenotype. Conversely, the PYK2 component activates RhoA in recipient tumor cells (68). This study provides the first evidence that fusion proteins can function as intercellular signaling factors to orchestrate PMN formation, offering a precise target for peptide-based disruption.

Parallel mechanisms reinforce this conditioning. OS-derived extracellular vesicles have been shown to package S100A11, a protein that specifically targets lung interstitial macrophages (69). Through the interaction with the deubiquitinase USP9X, S100A11 stabilizes JAK2/STAT3 signaling in macrophages. These activated macrophages subsequently secrete CXCL2 to recruit granulocytic myeloid-derived suppressor cells, creating a highly immunosuppressive barrier that facilitates tumor colonization (69). Furthermore, the cargo of exosomes differs critically between metastatic and non-metastatic cells. Only exosomes from highly metastatic OS lines induce TGF-β2 secretion in alveolar macrophages, suppressing their phagocytic capacity and effectively disarming the lung’s first line of immune defense (70).

### 6.2 The colonization phase: splicing variants and cytokine networks

Once OS cells arrive in the lung, the maintenance of metastasis requires sustained interplay with the immune stroma (**Figure 4**). Recent insights into RNA biology have highlighted the role of alternative splicing. U1 small nuclear ribonucleoprotein 70K, a splicing factor upregulated in metastatic OS, governs the alternative splicing of CD55 (decay-accelerating factor) (71–73). This splicing event facilitates a direct interaction between macrophages and tumor cells via the ADGRE5/CD55 signaling axis, promoting immune evasion and tumor expansion (71). This finding adds a layer of post-transcriptional regulation to the complexity of the metastatic niche.

Functionally, this interaction is amplified by cytokine loops. TAMs, which are more abundant in lung metastases than in primary tumors, secrete high levels of IL-8. This cytokine acts on OS cells to activate the FAK pathway, driving further proliferation and invasion (74). Simultaneously, TAMs induce EMT in tumor cells through the activation of the COX-2/STAT3 signaling cascade. The inhibition of COX-2 has been shown to repress EMT transcription factors and block metastasis, identifying this inflammatory axis as a crucial engine for metastatic colonization (75). Collectively, from the remote delivery of fusion proteins to the local splicing-mediated crosstalk, OS cells systematically corrupt pulmonary macrophages to transform the lung into a hospitable soil for metastasis.

## 7 Functional outcomes: from immune exclusion to chemoresistance

The ultimate consequence of the crosstalk between OS and macrophages is the acquisition of therapeutic recalcitrance. By reshaping the immune microenvironment, OS cells not only evade immune surveillance but also construct a biological shield against chemotherapy and foster a reservoir of cancer stem cells responsible for recurrence.

OS is classically characterized as an “immune-cold” tumor, but recent evidence suggests this is an active process of suppression rather than passive ignorance. The infiltration of CD163+ M2-type TAMs is directly correlated with a functionally exhausted T-cell phenotype (76). These tumor-infiltrating T cells co-express TIM-3 and PD-1, exhibiting diminished proliferation and impaired pro-inflammatory cytokine secretion (76). The depletion of CD163+ macrophages has been shown to restore T-cell vitality, proving that TAMs are the primary enforcers of this immunosuppression. This suppression is molecularly driven by OS-intrinsic factors. The overexpression of nuclear factor of activated T cells 3 in OS cells acts as a master regulator, simultaneously upregulating PD-L1 to directly inhibit T cells and promoting CXCL2 secretion by M0 macrophages (77). Similarly, Paired-like homeodomain transcription factor 1 downregulation leads to the exosomal release of LINC00662, which polarizes macrophages to secrete CCL22, further excluding effector cells (78). Additionally, the “don’t eat me” signal CD47, expressed on tumor cells, interacts with SIRPα on macrophages to block phagocytosis, a mechanism strongly linked to metastatic progression (79). The clinical relevance of this TAM-mediated immunosuppression has been corroborated in human cohorts. Multiplex imaging mass cytometry analyses of human osteosarcoma specimens have identified prognostic immunosuppressive macrophage subpopulations and highlighted their role in driving metastasis (80). These findings position macrophage reprogramming as a key contributor to metastatic progression and an important prognostic indicator in patients. However, this suppression is reversible. Innovative therapies, such as the engineered Salmonella strain VNP20009-CCL2-CXCL9, can reactivate the cGAS/STING pathway. This induces type I interferon secretion and promotes the recruitment of dendritic cells and T-cells, thereby repolarizing TAMs to an antitumor phenotype and demonstrating that the ‘cold’ TME can be reactivated (81).

Chemoresistance remains the major bottleneck in OS treatment. Paradoxically, neoadjuvant chemotherapy itself can induce resistance via macrophages. Chemotherapy stress stimulates macrophages to secrete IL-1β, which acts back on OS cells to reduce their drug sensitivity, creating a vicious cycle where treatment fuels protection (82). Beyond cytokines, sophisticated intracellular machinery is involved. MIF (macrophage migration inhibitory factor) activates the RAS/MAPK pathway in tumor cells; targeting MIF has been shown to restore sensitivity to cisplatin and doxorubicin (83). More intricately, the mitochondrial protein TIMM23 links metabolic stress to resistance. TIMM23 promotes macrophage M2 polarization via mitophagy; these macrophages then induce the expression of a novel TIMM23-PARGP1 fusion gene in OS cells (84). This fusion event enhances proliferation and directly confers chemoresistance, representing a newly identified therapeutic target. Furthermore, TAMs protect tumor cells from regulated cell death, specifically ferroptosis. M2-derived exosomes transfer Apoc1 to OS cells. Apoc1 binds to ACSF2, preventing its interaction with the deubiquitinase USP40. This leads to the ubiquitination and degradation of ACSF2, thereby blocking ferroptosis and allowing tumor cells to survive oxidative stress induced by chemotherapy (85).

The recurrence of OS is attributed to a subpopulation of OS Stem Cells (OSCs). Single-cell transcriptomics has unveiled a precise “lock-and-key” interaction between OSCs and TAMs. Macrophages secrete IGF-1, which binds to RARRES2 on OSCs. This IGF1-RARRES2 axis is essential for maintaining the stemness and heterogeneity of the tumor (86). Disruption of this communication induces differentiation and loss of stem-like properties, suggesting that targeting the macrophage niche is necessary to eradicate the root of the disease (86).

This malignant synergy of immune evasion, chemoresistance, and stemness maintenance represents the central barrier in OS therapy. Disrupting the cross-talk between tumor cells and macrophages—breaking this “co-conspiracy”—is essential to overcome therapeutic resistance and prevent recurrence.

## 8 Discussion

The analysis presented here underscores the limitations of a traditional ‘tumor-centric’ model, arguing instead that OS progression represents a dynamic co-evolution between malignant cells and TAMs. Our synthesis indicates that this synergy is not a passive reaction to inflammation but a precisely regulated program. Through integrated recruitment strategies, metabolic modulation, and epigenetic remodeling, OS cells co-opt the inherent plasticity of macrophages. This process effectively redirects TAMs from an anti-tumor phenotype to a state that actively supports tumor survival, chemoresistance, and metastasis.

A critical insight emerging from recent literature is the multi-layered nature of this communication. While classical cytokine signaling (e.g., CCL2, IL-10) remains relevant, the discovery of vesicular transport and fusion protein transfer represents a significant leap in our understanding of intercellular crosstalk. The identification of fusion proteins like Rab22a-NeoF1 and TIMM23-PARGP1 acting as intercellular signaling effectors—rather than just intracellular drivers—suggests that OS cells export their genomic instability to the microenvironment. Furthermore, the role of metabolic coupling—specifically the manipulation of amino acid transporters (LAT2) and lipid metabolism (LIPA)—demonstrates that OS cells impose a nutritional checkpoint, enforcing an M2-like state not just through signaling, but through the physiological constraints of nutrient availability.

Despite these profound mechanistic insights, the current preclinical evidence base presents several methodological limitations. Currently, most studies on OS-TAM crosstalk depend on *in vitro* cultures and PDX models. Because these models lack an intact human immune system, robust clinical validation remains limited. Furthermore, the species specificity of molecular regulators, such as ceRNA networks, complicates the extrapolation of murine data to human pathology. The lack of standardized protocols for exosome isolation also introduces technical variability that hinders clinical application. Addressing these gaps will require the integration of humanized *in vivo* models, target validation in larger patient cohorts, and the adoption of consensus methodologies.

Beyond these preclinical limitations, translating these findings into effective therapies presents its own clinical challenges. Current immunotherapies, such as immune checkpoint inhibitors, have shown limited efficacy in OS, likely because they fail to address the underlying immunosuppressive architecture maintained by TAMs. This clinical bottleneck is evident in the SARC028 phase 2 trial, where only 5% (1 out of 22) of osteosarcoma patients exhibited an objective response to the anti-PD-1 antibody pembrolizumab (87). Such clinical outcomes indicate that restoring T-cell activity is ineffective without concurrently dismantling the TAM-mediated immunosuppressive barrier. Merely depleting macrophages may be insufficient or even detrimental given their role in tissue homeostasis. Supporting this viewpoint, preclinical and early clinical data on macrophage-targeting agents highlight the limitations of macrophage depletion strategies. For instance, CCR2 inhibitors such as PF-04136309 have been shown to block monocyte recruitment in various solid tumors, yet often trigger compensatory recruitment of neutrophils or MDSCs, with CCL2 rebound potentially promoting metastasis upon discontinuation (88). In osteosarcoma models, CCL2-CCR2 axis inhibition modulates tumor-immune crosstalk but demonstrates restricted standalone efficacy (89). Similarly, while CSF-1R inhibitors (e.g., ABSK021/pimicotinib or PLX3397) achieve TAM depletion and direct anti-tumor effects in CSF-1R-overexpressing osteosarcoma preclinical models (90), their clinical translation as monotherapy has been remarkably unsuccessful (91). Clinical trials evaluating CSF-1R inhibitors as monotherapies have consistently demonstrated an exceptionally high failure rate, frequently yielding an objective response rate (ORR) of near 0% in advanced solid tumors and sarcomas (e.g., a 0% ORR in Phase II trials for pexidartinib in recurrent glioblastoma (92), and 0% for ARRY-382 and emactuzumab in advanced solid tumors (93)). For example, combining the selective CSF-1R inhibitor ABSK021 with standard-of-care chemotherapy has demonstrated enhanced synergistic anti-tumor activity *in vivo (*90), suggesting that modulating TAMs can improve traditional drug efficacy. Consequently, simple macrophage depletion is insufficient for durable tumor control, highlighting the mechanistic necessity for TAM re-education strategies. Instead, the future of OS therapy lies in “re-education” strategies—breaking the specific feedback loops identified herein. Agents that can reverse metabolic hijacking (e.g., LIPA inhibitors) or block specific innate immune checkpoints offer a viable avenue. Specifically, CD47 blockade has emerged as a strategy to reactivate macrophage-mediated phagocytosis. Evidence from preclinical xenograft models indicates that targeted inhibition of CD47 via specific antibodies suppresses the invasive capacity of OS cells and reduces spontaneous pulmonary metastasis by restoring the phagocytic clearance of tumor cells by TAMs (94). Furthermore, to achieve precise TAM re-education without systemic toxicity, nanoparticle-based delivery systems are being developed. Utilizing nanoscale carriers to deliver polarizing agents directly to the TME allows for a targeted M2-to-M1 phenotypic switch while minimizing off-target effects (91). Ultimately, dismantling this malignant synergy requires a combinatorial approach that targets both the tumor seed and the conditioned soil, moving beyond simple cytotoxicity to systemic immune normalization.
